# Therapeutic antibody targeting microtubule-binding domain prevents neuronal internalization of extracellular tau via masking neuron surface proteoglycans

**DOI:** 10.1186/s40478-019-0770-y

**Published:** 2019-08-07

**Authors:** Petronela Weisová, Ondrej Cehlár, Rostislav Škrabana, Monika Žilková, Peter Filipčík, Branislav Kováčech, Michal Prčina, Ľubica Wojčiaková, Ľubica Fialová, Tomáš Smolek, Eva Kontseková, Norbert Žilka, Michal Novák

**Affiliations:** 1 Department of Neuroimmunology, Axon Neuroscience R&D Services SE, Dvořákovo nábrežie 10, Bratislava, Slovak Republic; 2 Axon Neuroscience SE, Arch. Makariou & Kalogreon 4, Larnaca, Cyprus

**Keywords:** Alzheimer’s disease, Tau, Immunotherapy, Mechanism of action, Spreading, Heparan sulfate

## Abstract

Pathologically altered tau protein is a common denominator of neurodegenerative disorders including Alzheimer’s disease (AD) and other tauopathies. Therefore, promising immunotherapeutic approaches target and eliminate extracellular pathogenic tau species, which are thought to be responsible for seeding and propagation of tau pathology. Tau isoforms in misfolded states can propagate disease pathology in a template-dependent manner, proposed to be mediated by the release and internalization of extracellular tau. Monoclonal antibody DC8E8, binding four highly homologous and independent epitopes in microtubule-binding domain (MTBD) of diseased tau, inhibits tau-tau interaction, discriminates between healthy and pathologically truncated tau and reduces tau pathology in animal model in vivo. Here, we show that DC8E8 antibody acts via extracellular mechanism and does not influence viability and physiological functions of neurons. Importantly, in vitro functional assays showed that DC8E8 recognises pathogenic tau proteins of different size and origin, and potently blocks their entry into neurons. Next, we examined the mechanisms by which mouse antibody DC8E8 and its humanized version AX004 effectively block the neuronal internalization of extracellular AD tau species. We determined a novel mode of action of a therapeutic candidate antibody, which potently inhibits neuronal internalization of AD tau species by masking of epitopes present in MTBD important for interaction with neuron surface Heparan Sulfate Proteoglycans (HSPGs). We show that interference of tau-heparane sulfate interaction with DC8E8 antibody via steric hindrance represents an efficient and important therapeutic approach halting tau propagation.

**Electronic supplementary material:**

The online version of this article (10.1186/s40478-019-0770-y) contains supplementary material, which is available to authorized users.

## Introduction

Alzheimer’s disease (AD) and related neurodegenerative disorders are the leading cause of dementia worldwide and their prevalence is rapidly increasing. Despite intensive research and pharmaceutical progress, understanding the trigger or defining the cause(s) of Alzheimer’s disease are still among the main scientific challenges of neurodegeneration research. Neurofibrillary pathology formed by abnormal forms of microtubule-associated protein tau has been recently emerged as a dominant factor in AD pathogenesis, and has been identified as the main correlate of cognitive decline in AD and other tauopathies. Current research of tauopathies focuses on neurofibrillary lesions, a common pathological hallmark composed of deposits of tau protein in aggregated form [58]. While physiologically functional tau is an unfolded monomeric protein responsible for microtubule stabilisation, neuronal outgrowth and regulation of organelle transport in axons [61], pathological forms of tau which include truncated monomers, oligomers and higher order aggregates are toxic to neurons [77]. We and others have found that misfolded tau protein is directly responsible for synaptic impairment, neuroinflammation, blood brain barrier abnormalities and cognitive or motor deficits in rodent animal models [5, 39, 43, 52, 67, 105, 107].

Progressive development of tau pathology in AD is dependent on transmissibility of aggregated tau protein throughout the brain, thus leading to a characteristic pattern of spatiotemporal/hierarchical spreading [17]. The tau pathology manifest in the trans-entorhinal cortex and spreads in stereotypical manner via neuro-anatomically connected brain areas [8, 12, 40, 55]. Aggregated and misfolded tau protein variants have the ability to propagate disease pathology in a template-dependent manner, which is termed as seeding [65]. Interestingly, misfolded tau aggregates from Alzheimer’s disease (AD) and other tauopathy brains may vary in their seeding and propagation propensity [22, 35, 47, 53, 76]. The propagation of tau throughout the CNS occurs via cell to cell transfer of misfolded tau forms, released by neurons to extracellular milieu which subsequently enter healthy adjoined neurons and induce further fibrillization [9, 26, 28, 34, 48, 95]. Therefore, promising therapeutic intervention consists of targeting extracellular pathogenic tau variants, which are the mediators of seeding and spreading of tau pathology. Indeed, functional cellular assays testing antibodies mapping various epitopes on tau protein are currently used for screening for the best therapeutic antibody candidate based on their ability to block tau internalization and seeding [15, 68, 88, 102], with antibodies targeting predominantly mid-domain of tau showing the maximum potency. Sulfated heparan proteglycans (HPSGs) on the cell membrane are required for the uptake and secretion of misfolded tau species [27, 37, 46, 74, 84]. Therefore, therapeutic interventions with capability to block tau-HSPGs interactions and thus preventing spread of tau pathology in the brain may be an important factor potentially contributing to desired clinical benefit.

Previously, we have developed a unique anti-tau antibody DC8E8 interacting with four highly homologous epitopes present in the microtubule-binding domain (MTBD) of tau protein, with its binding mode ensuring the effective blocking in β-structure formation [49]. Additionally, DC8E8 antibody exhibited higher affinity for pathologically truncated tau over physiological tau and effectively protected the brain from neurofibrillary pathology in vivo [49].

In the present study, we provide a detailed mechanistic insight into the therapeutic mode of action of DC8E8 antibody at neuronal level. Our data revealed predominant extracellular mechanism of action of DC8E8 and its strong ability to recognise all misfolded tau aggregates varying in size, conformation and origin, ensured by its binding epitope to tau. Importantly, DC8E8 proved to be a potent suppressor of internalization and seeding of tau aggregates into primary neurons, thus effectively intercepting spreading of tau to neihgbouring neurons. Binding of tau aggregates to sulfated heparan proteoglycans (HSPGs) on neuronal surface requires heparin binding motifs that are localised in close vicinity to DC8E8 binding epitopes. Our results suggest that intercepting the binding of tau seeds to heparan sulfate surface proteins, with DC8E8 antibody targeting tau in the microtubule-binding domain is an effective immunotherapeutic strategy.

## Material and methods

### Antibodies

Monoclonal antibody AT8 recognizing a phosphorylated tau pS202-pT205-pS208 and tau monoclonal antibody HT7 recognising the epitope 159–163 of human tau protein were purchased from Thermo Fisher Scientific, antibody DC190 mapping tau 368–376 was purified from hybridoma supernatant and conjugated to horse radish peroxidase. Antibodies DC8E8 [49] and DC25 (epitope 347–353) were affinity purified from serum-free hybridoma supernatant. Indiferent monoclonal antibody DC51 (IgG1 isotype) was used as unrelated control (specifically binds to surface glycoprotein of rabies virus) [56]. Monoclonal anti-tau antibodies AX004/IgG1 and AX004/IgG4, humanized version of anti-tau monoclonal antibody DC8E8 [49] were produced in Expi-CHO cells (Thermo Fisher Cat. No. A29133). The activity of purified antibodies was verified by ELISA, Western blot and immunohistochemistry.

### DC8E8 vaccine administration

DC8E8 vaccine was administered in 2 weeks intervals starting at the age of 6 weeks and continuing until the age of 6 months. Purified antibody DC8E8 prepared in PBS was injected intraperitoneally (1 mg of antibody/per dose/200 μl) into transgenic mice R3m/4 expressing human truncated tau (151–391/3R) under the control of the mouse Thy1 promoter, with tau pathology predominantly located in the brainstem [107]. An additional set of R3m/4 animals was injected in the same vaccination scheme (timing and dosing) with unrelated antibody DC51 (anti-rabies IgG1), thus representing the control. Then animals were sacrificed and their brainstems were subjected to immunohistochemical and biochemical quantification of tau pathology. The experiment was carried out in accordance with the Slovak and European Community Guidelines, approved by the State Veterinary and Food Administration of the Slovak Republic.

### Quantitative immunohistochemistry of mouse brain tissue samples

Mouse were anesthetized and perfused intra-cardially for 1 min with phosphate-buffered saline (PBS), followed by 3 min perfusion with 4% paraformaldehyde (PFA) in PBS (4% PFA, pH 7.2). Brain were transferred for post fixation for 24 h in 4% PFA, then transferred to PBS, embedded in paraffin and serially cut using Leica RM 2255 microtome into 8 μm thick sagittal brain sections. Immunostaining was performed using the standard immunohistochemistry staining procedure. Briefly, brain sections were treated with 80% formic acid (40 s) followed by heat pretreatment for 20 min in antigen retrieval solution (Retrieval 2100, Aptum, Southampton, UK). The sections were blocked with Aptum section block (Aptum, Southampton, UK) followed by incubation with mouse monoclonal primary antibody (Anti -human phospho tau AT8, 1:1000, ThermoScientific, IL, USA) overnight at 4 °C and were immunostained using the standard avidin-biotin-peroxidase method (Vectastain ABC kit) with VIP as chromogen (VIP kit, Vector Laboratories, Burlingame, CA, USA). After mounting, sections were evaluated using Olympus BX51 microscope. Neurofibrillary tangles were counted in two parallel sections from each mouse brain. Quantification of structures was carried out by investigators blinded to the treatment status of the mice.

### Preparation of sarkosyl-insoluble tau protein (2p fraction)

Brainstems of transgenic animals R3m/4 were subjected for biochemical analysis of sarkosyl-insoluble tau proteins. The sarkosyl-insoluble fraction was extracted according to published protocol [32]. Frozen tissues were homogenized for 30 s in tenfold weight excess of ice-cold extraction buffer (20 mM Tris, pH 7.4, 800 mM NaCl, 1 mM ethylene glycol tetraacetic acid), 1 mM ethylenediaminetetraacetic acid, 10% sucrose, containing protease inhibitor (Roche Diagnostics) and phosphatase inhibitor cocktail (Sigma Aldrich). The homogenates were centrifugated at 20,000 g for 20 min at 2 °C. The supernatants designed 1 s were transferred into clean tubes. Next, solid sarkosyl (N-lauroylsarcosine sodium salt; Sigma-Aldrich) was added to the 1 s supernatant to achieve 1% concentration and then stirred for 1 h at room temperature (RT). Samples were then centrifuged at 100,000 g for 1.5 h at RT. Pellets (designated 2p) were gently rinsed with 1 ml of the sarkosyl extraction buffer and spun at 100,000 g for 20 min at RT. The pellets (sarkosyl-insoluble tau fractions) were resuspended in 6 M Guanidine for ELISA or in PBS for Western Blotting analysis to a final volume representing the 1/25 volume of the 1 s fraction followed by 5 min sonication and stored at − 20 °C. The sarkosyl-insoluble 2p tau fractions were also isolated from human AD brain tissue using the same procedure as described above. Human brain samples (transentorhinal cortex, NFTs rich Braak stage VI, AD sporadic and AD familial with a missense mutation of PSEN1 (Thr116Asn) [85]) were obtained from Newcastle Brain Bank and Slovak Brain Bank in accordance with ethical approval.

### Biochemical quantification of sarkosyl-insoluble tau using sandwich ELISA

Sarkosyl-insoluble tau fractions (2p) were subjected for biochemical quantification using sandwich ELISA assays (AT8/DC190 for transgenic animal efficacy study; DC25/DC190 for neuronal internalization in vitro experiments). A 96-well ELISA plate (Nunc Medisorp, Denmark) was coated with 2 μg/ml AT8 antibody (efficacy studies) or DC25 antibody (AD tau neuronal internalization) in PBS overnight (16–18 h) at 4 °C. The plate was washed five times with PBS buffer supplemented with 0.075% Tween 20 (PBST) followed by blocking with PBST buffer for 1 h at RT. Guanidine hydrochloride samples of sarkosyl-insoluble 2p fractions were diluted 50-fold with PBST buffer (in duplicates). For standard curves, recombinant pathogenic tau 151–391/4R (in vitro phosphorylated, affinity purified) was used, in 2 fold dilution steps in PBST buffer. The plate with tau standard and sarkosyl-insoluble tau fractions was incubated 90 min at 37 °C, followed by washing with PBST for five times. As detection antibody DC190-HRP at 1: 15000 dilution in PBST (0,3 μg Ab/ml) was used and incubated for 60 min at 37 °C, followed by washing with PBST for five times. Next, substrate Colorburst Blue (TMB/peroxide substrate, ready to use; Alerchek USA) was added to the plate and incubated for 20 min in dark. The reaction was stopped by adding of 0.25 M H_2_SO_4_. The 450 nm absorbance was measured and plotted against the protein concentration of tau 151–391/4R standard. Concentrations of AT8-positive (efficacy study) or DC25-positive tau (neuronal internalization) in samples was obtained based on extrapolation from the standard calibration curve.

### Expression, purification and fibrillization of recombinant tau protein

Truncated tau 297–391/4R, dGAE (t-tau; numbering according to the longest human tau isoform 2N4R) was expressed in *Escherichia coli* strain BL21(DE3) (Sigma-Aldrich, St. Louise, Missouri, United States) from a pET-17 expression vector and purified from bacterial lysates as described previously [16], except the anion-exchange chromatography step was omitted and size-exclusion chromatography was performed in PBS-argon (137 mM NaCl, 2.7 mM KCl, 10 mM Na2HPO4, 2 mM KH2PO4, pH 7.4) (AppliChem GmbH, Darmstadt, Germany). Purified tau protein was stored in PBS-argon in working aliquots at − 70 °C. The purity of tau protein was subsequently verified by gradient SDS gel electrophoresis (5 to 20% gel), Coomassie blue staining and Western blot analysis with DC25 antibody (AXON Neuroscience SE, Bratislava, Slovakia), recognizing residues 347–354 of the longest human tau isoform 2N4R). In vitro fibrillisation of recombinant truncated tau protein (100 μM) was carried out using heparin (Sigma-Aldrich, St. Louis, Missouri, United States) as an inducer at a concentration 240 μM tau + 60 μM heparin in PBS (137 mM NaCl, 2.7 mM KCl, 10 mM Na2HPO4, 2 mM KH2PO4, pH 7.4). The reaction was performed for 24 h at 37 °C. After incubation, tau aggregates were sonicated for 2 min at 20% power output using an MS72 probe of a Bandelin Sonopuls Sonifier (Bandelin, Berlin, Germany). Subsequently, 1 μM aliquots were stored at − 70 °C. The oligomerization of the tau protein was verified by non-reducing SDS-PAGE gel electrophoresis and quantitative thioflavin T (ThT) fluorescence spectroscopy with excitation at 450 nm and emission at 510 nm.

### Fluorescence labelling

The fluorescently tagged tau protein (after fibrillization) was prepared by labelling with Alexa Fluor™ dyes (Invitrogen, Carlsbad, California, United States) according to the manufacturer’s recommendations. The labeling was carried out at pH 6.5 for preferential labeling of N-terminus. Succinimidyl esters of Alexa Fluor™ 488 and Alexa Fluor™ 594 (ThermoFisher) were dissolved in anhydrous DMSO (Molecular probes) and mixed with tau proteins (in PBS) in 5:1 M ratio. The mixture was incubated at RT for 1 h with 400 rpm shaking. The unreacted dye was subsequently separated from the labeled tau protein using Zeba 7 k MWCO desalting spin columns (Thermo Fisher Scientific) equilibrated with PBS. Labeled truncated tau (297–391/4R) and human brain-derived AD Tau (2p sarkosyl-insoluble fraction) were incubated at 37 °C with 700 rpm shaking for 2 days. The size distribution was monitored using DLS with DynaProNanoStar (Wyatt technologies). The sample was centrifuged for 5 min at 5000 g prior to DLS measurement.

### Dynamic light scattering (DLS)

Tau protein samples (10 μl) were centrifuged at 5 000 g for 5 min at 25 °C, transferred into a 4 μl disposable cuvette (Wyatt Technology) and measured in a DynaproNanoStar instrument controlled by Dynamics software v. 7.7.0.125 (Wyatt Technology). Measurements were performed in 1 second acquisition time averaged 10-times except for truncated tau dGAE monomer, which was measured using 10 s acquisition time. Data from at least five individual measurements of dynamic light scattering (DLS) per sample were evaluated by the Dynamics software v. 7.8.0.26. To cull the acquisitions influenced by dust or irregular particles, an automatic filtering of autocorrelation functions was applied with an individual limit for baseline threshold and maximal allowed sum-of-squares (SOS) error for cumulants fit. After filtering, at least 65% of original data remained for analysis. To determine the size distribution of protein preparations, DLS autocorrelation data were subjected to a regularization analysis by Dynals algorithm. Final graphs were prepared in Prism 6 software (GraphPad).

### Infrared spectroscopy

Infrared spectra were collected on ThermoScientific Nicolet iS50R Research FTIR Spectrometer equipped with a DTGS detector (Thermo Fisher Scientific). The instrument and sampling accessory were continuously purged with water and CO_2_ free air. One μl of sample in PBS was loaded into a ConcentratIR2 Multiple Reflection ATR (Harrick Scientific Products) adopting a silicon element with a nominal incident angle of 30° and eleven reflections. The sampling plate was sealed and the sample drop dried under flow of dry air. After vanishing of liquid water absorption bands, the flow of dry air was stopped and 32 scans were collected at 4 cm^− 1^ resolution within a spectral range of 650–4000 cm^− 1^ wavenumbers. Spectra were collected using zero-filling factor 2, Happ-Genzel apodization, Mertz phase correction, aperture 160, samples gain 4, optical velocity 0.4747 cm.s^− 1^. Reference spectra were recorded under identical conditions with empty ATR sampling plate and were subtracted from the protein-sample spectra. Spectra were further baseline-corrected and processed with ATR advanced correction as implemented in OMNIC software v. 9 (Thermo Fisher Scientific).

### Kinetics of truncated tau (297–391/4R) aggregation – Thioflavin T (ThioT) fluorescence spectrometry

Detection of tau filaments assembly-aggregation of recombinant tau protein was monitored by ThT fluorescence. 40 μl of 350 μM truncated-tau 297–391 (dGAE) with 20 μM ThioflavinT (Sigma) was incubated in black solid polystyrene 384 wells Greiner BioOne plate in the absence and presence of heparin (tau-heparin concentration ratio 4:1). The fluorescence was measured using the Fluoroskan Ascent FL (Labsystems) every 10 min with excitation at 450 nm and emission at 510 nm. The plate was shaken at 720 rpm and incubated at 37 °C. The aggregation kinetics was monitored for 4 days.

### Immunoprecipitation of native, sarkosyl-insoluble AD tau with DC8E8 antibody

The sarkosyl resistant 2p tau fraction was isolated from 1 g of AD human brain sample using the procedure outlined above for mice brain tissue. The brain was examined by immunohistochemistry and tau pathology found to correspond to Braak stage VI. The 2p pellet fraction was re-suspended in 1 ml of PBS (supplemented with 50 mM NaF, 1 mM Na3VO4 and the cocktail of protease inhibitors Complete® without EDTA (Roche)) by sonication for 2 min on ice using a Bandelin Sonopuls HD2200/UW2200 equipped with a MS72 probe, at 20% duty cycle with the output set at 20% (Bandelin Electronic, Germany). The resulting suspension was split into two 500 μl portions and each portion received 25 μg of one of two purified antibodies: either DC8E8 or a control antibody DC51. The suspensions were incubated with the antibodies with head-over-tail rotation at 6 °C for 2 h. In order to isolate the antibody-disease tau complexes, 50 μl of the 50% suspension of Protein G Mag Sepharose beads (GE Healthcare), equilibrated in PBS+ 0.01% Igepal CA-630 (SIGMA), were added to each of the two mixtures of sarkosyl-insoluble AD tau and antibodies. The immunoprecipitation mixtures were further incubated at 6 °C for 1 h. The beads in each of the incubation mixtures with bound antibody-tau complexes were harvested using magnet and washed three times with 150 μl of PBS (supplemented with 50 mM NaF, 1 mM Na3VO4, 0.02% IGEPAL CA-630 (SIGMA) and the cocktail of protease inhibitors Complete® without EDTA (Roche)) and once with 150 μl of PBS only. The bound antibody complexes were eluted from the beads by three consecutive 5-min incubations in 100 μl of 200 mM formic acid pH 2.7. The eluates were pooled, lyophilized, the proteins dissolved in SDS-PAGE sample loading buffer, separated on 12% SDS-PAGE gels, transferred onto nitrocellulose membranes and the tau proteins detected by incubation with the pan tau antibody DC25 conjugated to HRP (Kementec, Denmark). The blots were developed with a SuperSignal West Pico Chemiluminescent Substrate system (Pierce, U.S.A) and the signals detected using a LAS3000 imaging system (FUJI Photo Film Co., Japan).

### Cortico-hippocampal neurons, viability, viral transduction and cellular physiological functions

Rodent cortico-hippocampal neurons were prepared and cultured as described, with minor modifications [10]. Neocortes were isolated from pregnant female mice C57BL6N on embryonic day 16–18 using 10% Ketamidor and 10% Xylariem as lethal anesthesia. Brains of embryos were dissected out, placed in ice-cold sterile L-15 medium free of L-glutamine (PAA) and meninges were removed. Cerebral cortices with hippocampi were isolaed, chopped into small pieces and incubated with 0.25% Trypsin-EDTA for 10–15 min at 37 °C. After incubation, trypsinisation was inhibited by adding media with inactivated serum. The cells were dissociated by gentle pipetting with subsequent centrifugation at 300 g for 5 min. Cortico-hippocampal neurons were triturated with glass Pasteur pippete in fresh plating media (DMEM supplemented with 10% (v/v) fetal calf serum, 2 mM L-glutamine and 100 units/mL penicillin/streptomycin (all from Life Technologies Invitrogen, Carlsbad, California, United States). Cells were plated onto Poly-D-lysine coated 6 well plates or glass Nunc Lab-Tek Chambers (Thermo Fisher Scientific) at a density of 2.10^5^ cells/cm^2^ (unless indicated otherwise) and and cultivated at 37 °C, 5% CO2 in a water-saturated atmosphere. After 24 h plating media was exchanged for Neurobasal Media containing 2% B27, 2 mM L-glutamine and gentamycin 10 μg/mL. Experiments were carried out after 2 ± 5 days in vitro (DIV). Transfection of primary neurons with fluorescently labelled antibody DC8E8 Alexa Fluor 546 (30 μg) was performed with MaxCyte Flow Electroporation™ Technology according to the manufacters instructions and cells were examined for viability/nuclear morphology (Hoechst 33258 Sigma, 1 μg/ml; Trypan blue Sigma) and physiological functions (ENLITEN ATP Assay System Bioluminescence Detection kit; Promega) 24 h later. Alternatively, primary neurons 1–2 DIV were transduced with virus containing human truncated tau - AAV9-hSynapsin1-tau (151–391)-P2A-mCherry WPRE (Vector Biolabs). Two to three days after AAV viral transduction (9.1 × 10^13^ GC/ml) neurons were examined for t-tau (151–391/4R) protein expression with immunocytochemistry and western blotting. All procedures involving animal work were in accordance with ethical standards and approval from the State Veterinary and Food Committee of Slovak Republic and the number of sacrifised animals recorded.

### Neuronal tau internalization assay

Truncated tau (297–391/4R) and human brain-derived AD tau (transentorhinal cortex from two sporadic AD patients and one familial AD case [85], both NFT rich Braak stage VI) fluorescently labelled with Alexa Fluor 488 or Alexa Fluor 594 were diluted in neuronal conditioned media at the concentration corresponding to 100–200 nM for monomeric tau protein in combination with the following antibodies: unrelated control antibody DC51 [56], mouse monoclonal DC8E8 (mDC8E8), humanized DC8E8 (AX004/IgG1; AX004/IgG4) and their isotype controls IgG1/IgG4 (BioLegend), all of concentration of 1 μM and incubated for 30 min at 37 °C. Pre-formed tau-antibody complexes were added to neurons for 24 h. For experiments where heparin was used (Tinzaparin sodium, Sigma-Aldrich, which is a LMWH), fluorescently labelled tau aggregates were preincubated with heparin (5 μM) for 30 min and heparin-AD tau complexes were applied to neurons for 16–20 h. Neurons were washed three times with pre-warmed PBS, followed by mild trypsinisation (0.06% trypsin-EDTA 2–3 min) to remove cell surface bound tau and dissociate neurons into single cells. Next, neurons were processed live for flow cytometry measurements to quantify the amount of fluorescently labelled internalized tau (BD LSRFortessa™ II cell analyzer). Neurons were gated using forward and side scatter to remove cellular debris. Measurements were recorded as mean fluorescent intensity of Alexa Fluor 488 or Alexa Fluor 594. Alternatively, neurons grown on glass Nunc Lab-Tek Chambers (Thermo Fisher Scientific) were imaged for fluorescence AF488 labelled tau protein (excitation at 488 nm and emission at 525 nm) with a 20× objective LSM 710 confocal microscopy and examined for intracellular localisation by fluorescent labelling of lysosomes with fluorescent dye LysoTracker (75 nM, 45 min Thermo Fisher Scientific). Typically, experiments were performed in triplicates from minimum of three independent experiments and 10 000 cells for each well were quantified by flow cytometry. Data were analysed using Prism Software (GraphPad).

### Immunocytochemistry

Rodent cortico-hippocampal neurons were cultured on cover glass pre-coated with Poly-D-lysine at a density of 1 × 10^6^ cells per milliliter and cultivated for 48 h. Neurons were treated with fluorescently labelled (Alexa Fluor 488 or Fluor 594) sarkosyl-insoluble AD tau (2p, 100 nM) only or in combination with control and DC8E8/AX004 antibody (1 μM) and cultivated for 20 h in conditioned Neurobasal media at 37 °C, 5% CO2. Next day neurons were washed with pre-warmed PBS and mild trypsin (0,06% trypsin-EDTA, 3 min), fixed with 4% PFA-PHEM, pH 6.9 (60 mM PIPES, 25 mM HEPES, 10 mM EGTA, 2 mM MgCl_2_, PFA) for 12 min. Neurons were permeabilized with 0.1% Triton X100 in PBS (PBS-T) and blocked with 5% BSA in TBS-T. The cells were then incubated with anti-heparan sulfate antibody (10E4, AMSBIO 1:100) for 1 h or, in a different experiment with HT7 antibody (epitope against human tau, MN1000, Thermo Fisher Scientific 1:500), washed and incubated with secondary antibody goat anti-mouse Alexa Fluor 488 (Invitrogen). The samples were mounted in FluoroshieldTM medium with DAPI (Sigma-Aldrich). Images were captured by LSM 710 confocal microscope (Zeiss, Jena, Germany).

### Western blot analysis of fibrillized and sarkosyl-insoluble tau

Samples of fibrillized truncated tau 297–391/4R (1 μg) and human-derived AD tau fractions (6 μl) from sarkosyl isolations (2p, 1 s and 2 s) were mixed with SDS sample loading buffer and heated at 95 °C for 5 min. Each sample (6 μl) was then loaded onto 5–20% gradient SDS polyacrylamide gels and electrophoresed in a Tris-glycine-SDS buffer system for 40 min at 25 mA. Proteins were transferred to nitrocellulose membrane and, after blocking in 5% fat-free dry milk in PBS for 1 h at room temperature, the membrane was incubated for 1 h with pan-tau mAb DC25 and with therapeutic antibody DC8E8. After washes, HRP-conjugated goat anti-mouse Ig (Dako Denmark) diluted 1:3,000 in PBS was used as a secondary antibody. Blots were washed and developed using ECL chemiluminescence detection (GE Healthcare), detected with SuperSignal West Pico Chemiluminescent Substrate (Pierce Biotechnology), and imaged using a FujiFilm LAS-3000 imaging system (Fuji).

### Statistical analysis

Data are presented as means ± SEM. To compare two groups, the Mann–Whitney *U* test or an unpaired *t*-test was applied. For statistical comparison of more groups, one-way analysis of variance ANOVA and post hoc Tukey’s test were used using Prism software v. 7 (GraphPad Software, Inc., San Diego, USA). Differences were considered significant at the level of *p* < 0.05.

## Results

### Antibody targeting microtubule binding region (MTBR) provides neuroprotection against tau pathology in transgenic animals

**Fig. 1:**
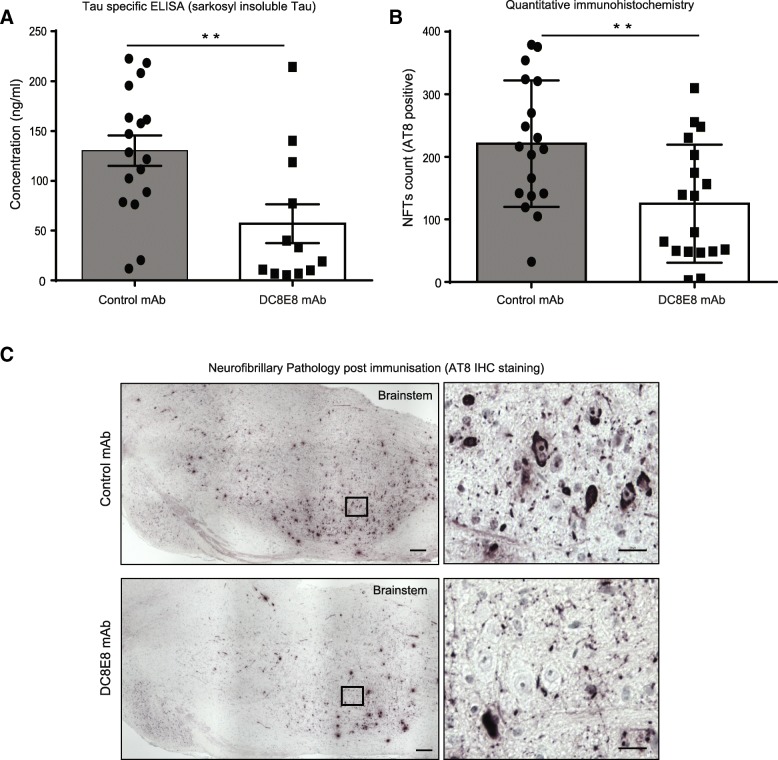
In vivo efficacy of tau antibody DC8E8 targeting microtubule binding domain in the brains of transgenic mice. **a** Biochemical quantification of sarkosyl-insoluble tau protein extracts (2p) isolated from brainstem of transgenic mice treated with irrelevant control DC51 (*n* = 17) vs immunotherapeutic DC8E8 antibody (*n* = 12) evaluated using sandwich AT8/DC190 HRP ELISA. Data are presented as mean ± SEM. p* ≤ 0.01 with statistically significant reduction in the induction of tau pathology in DC8E8 treated animals in comparison to animals treated with control antibody. **b** Quantitative immunohistochemistry of mouse brain tissue samples. Neurofibrillary tangles were counted in two parallel sections of the brainstem from each mouse (control *n* = 18, DC8E8 n = 18). Quantification of AT8-positive NFTs was carried out by investigators blinded to the treatment status of the mice. Data are presented as mean ± SEM. p** = 0.0054 with statistically significant reduction in the formation of NFTs in DC8E8 treated animals in comparison to animals treated with control antibody DC51. **c** Representative images of pathological structures consistent with neurofibrillary tangles (NFTs) positive for AT8 immunohistochemical staining in transgenic mice R3m4 expressing human truncated tau (151–391/3R) treated with irrelevant control (DC51) or DC8E8 antibody. AT8 immunostaining was robustly decreased in mice after immunization with DC8E8 antibody in comparison to control antibody. Scale bars: 200 μm; right panel 20 μm

A growing body of recent experiments revealed that the antibody epitope rather than its affinity to pathological tau proteins is an important factor in mediating therapeutic efficacy [15, 88]. Previously, we generated a murine monoclonal DC8E8 antibody with the strong potential to abolish pathological tau-tau interaction by targeting four separate epitopes localized in human tau protein microtubule binding regions MTBR1–4 [49]. In the biosensor SPR analysis, antibody DC8E8 exhibits nanomolar afinity with nearly an order of magnitude stronger binding to pathological truncated tau protein than to physiological full-length tau [44]. In order to determine the therapeutic potency of DC8E8 antibody in vivo, we utilized transgenic mice expressing pathogenic non-mutated human truncated tau (151–391/3R) with the ability to induce neurofibrillary pathology similar to human NFTs located predominantly in the brainstem [73, 107]. Transgenic mice were treated with mDC8E8 or unrelated control antibody DC51 of the same isotype (IgG1) biweekly (40 mg/kg) for 5 months. In line with our previous results [49], we confirmed the strong ability of this therapeutic antibody to reduce the development of neurofibrillary pathology measured here with independent method of sarkosyl-insoluble tau-specific ELISA (Fig. 1 a). A significant suppression of the development of tau pathology in the brainstem of transgenic mice treated with DC8E8 in contrast to animals treated with control antibody DC51 was measured (with ~ 70% decrease in the amount of sarkosyl-insoluble tau). Quantitative histopathological examination of brains from transgenic animals treated with DC8E8 antibody revealed reduced accumulation of AT8 positive pretangle and tangle bearing neurons when compared to mice treated with control antibody (Fig. 1 b, 1c), and supported the high efficacious effect of DC8E8. Biochemical and histopathological measurements, thus demonstrated a robust therapeutic effect of DC8E8 antibody targeting four separate conformational tau epitopes in MTBR, as shown by a strong in vivo supression of tau pathology development in the brains of transgenic mice*.* Similarly, AADvac1 active vaccine, currently in clinical trials, that targets one of DC8E8 epitopes and elicits production of antibodies with the same tau binding properties as DC8E8 [73], proved to be efficacious with the capability to reduce the development of tau pathology in transgenic animals to similar extent [49, 50].

### DC8E8 antibody acts via extracellular mechanism and does not influence viability of neuronal cells

**Fig. 2:**
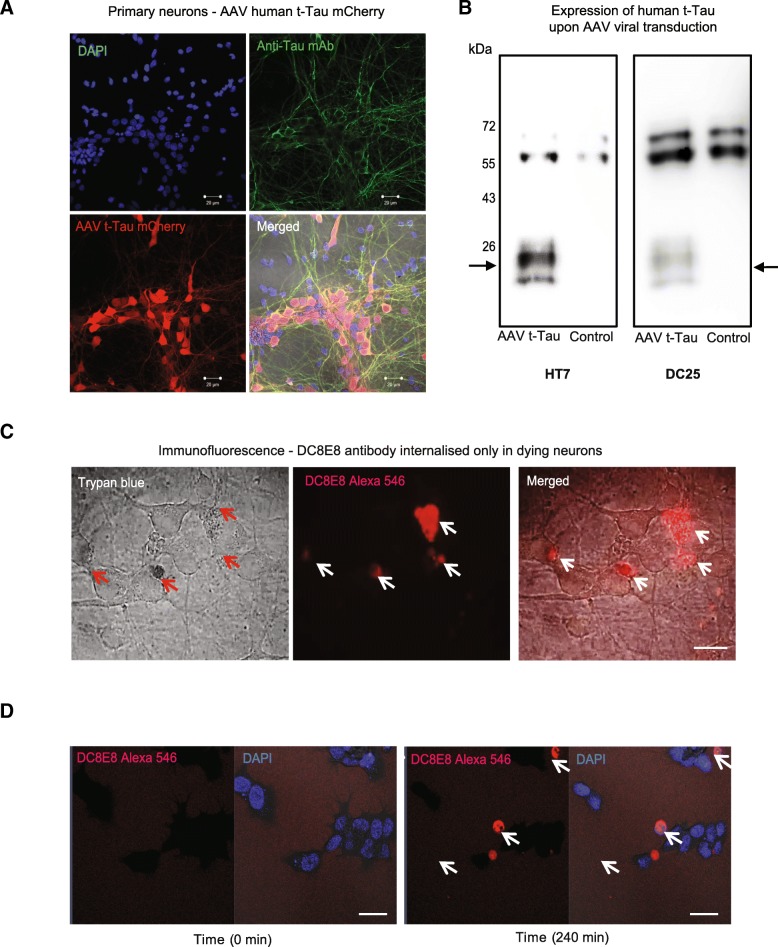
Monoclonal antibody DC8E8 requires no uptake into primary neuronal cells with pathogenic truncated tau. **a** Characterization of primary cortico-hippocampal neurons transduced with pathogenic AAV human truncated tau linked to mCherry (red). Forty eight hours after AAV viral transduction neurons were examined for truncated tau expression (151–391/4R) by immunocytochemistry with anti tau antibody (DC190 recognising epitope 368–376, green). Merged image confirmed substantial co-localization between the AAV transduced t-tau and additional pan tau staining. **b** Three days after AAV transduction primary neurons were screened for t-tau protein expression by Western Blot utilizing 2 different anti tau antibodies (HT7; DC25 recognising 347–353). SDS-PAGE and immunoblotting in both cases show expression of the 25 kDa human truncated tau protein in neuronal cells as indicated (arrows). **c** Primary neurons cultured on glass with confirmed AAV transduced t-tau protein were examined for viability with trypan blue. Subsequently, neurons were subjected to addition of DC8E8 antibody. Immunocytochemical labelling of DC8E8 showed that therapeutic antibody was detected and localized only in trypan blue positive neurons (dying neurons) as indicated by arrows. **d** Human neuroblastoma cells SHY5Y expressing human truncated tau protein (151–391/3R), were cultured on glass Labtec dishes and stained live for condensed nuclei (Hoechst 33358 1 μg/mL). Next, cells were subjected to an addition of fluorescently labelled humanized version of DC8E8/AX004 Alexa Fluor 546 (red) in media (1 μM ~ 168 μg/mL) and monitored over time with LSM 710 confocal microscopy for up to 4 h. AX004 antibody was not internalized in neuronal cells with t-tau expression, only in dying cells with condensed nuclei (Hoechst) as indicated with arrows. Experiments were repeated 3 times with similar results obtained. Scale bars, 10 μm

Previous pre-clinical evidences from Aβ and tau immunotherapies demonstrated that therapeutic antibodies can cross blood-brain barrier and accumulate in the brain to mediate their effects [2, 7, 30, 31, 75, 81]. To gain a mechanistic insight into the therapeutic mode of action of DC8E8 antibody at neuronal level, we first tested (in ex vivo conditions) whether antibody DC8E8 is internalized into neurons containing diseased tau proteins to mediate their clearance. Murine cortico-hippocampal primary neurons derived from embryonic day 17.5 pups were infected with adeno-associated virus containing pathogenic truncated tau (t-tau, 151–391/3R) [24, 107] linked to red fluorescent protein mCherry. AAV-mediated transduction of pathogenic tau was evaluated by immunocytochemistry using pan tau antibody (DC190) in neurons cultured for 5 DIV. Tau protein expression was confirmed by robust colocalization of AAV-transduced neurons positive for mCherry and tau antibody (DC190) staining in cell bodies and neurites, as well as by immunoblotting with a band of size specific for truncated tau (25 kDa), (Fig. 2 a, b). To monitor the uptake of DC8E8 antibody into tau diseased neurons, we performed immunocytochemistry to examine the intracellular localisation of DC8E8 (Fig. 2 c). Interestingly, we observed the intracellular presence of DC8E8 only in neurons with compromised plasma membrane integrity (Fig. 2 c). To confirm that DC8E8 antibody is not internalized in tau diseased neuronal cells, we utilized an additional model of neuroblastoma cells SH-SY5Y expressing t-tau (151–391/4R). Confocal live cell imaging with fluorescently labelled DC8E8 antibody (red) added into the cell culture media of tau expressing cells showed comparable results. We found that antibody was only detected intracellularly in tau expressing cells with condensed nuclei, not in live neuronal cells. Taken together, our findings suggest that the predominant therapeutic effect of DC8E8 antibody is extracellular. This finding is in a line with previous reports of other immunotherapies targeting tau protein [44, 102].

**Fig. 3:**
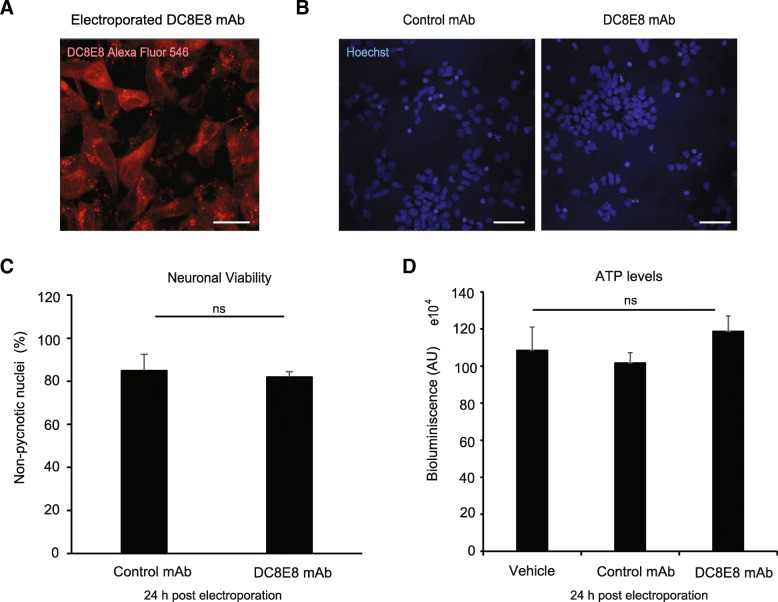
Mouse and humanized antibodies mDC8E8/AX004 do not influence neuronal viability and physiological ATP production. **a** Representative image of human neuroblastoma cell line SHY5Y with internalized fluorescently labelled AX004 Alexa Fluor 546 antibody (60 μg) 24 h post electroporation. Microtubules were decorated with AX004 Alexa 546, thus confirming the specific intracellular binding site. **b** Fluorescent image of rodent primary neurons stained with Hoechst (c = 5 μg/mL, 30 min incubation live cells at 37 °C) 24 h post electroporation with unrelated control mAb DC51 or AX004 mAb. Scale bar, 20 μm. **c** Quantification of neuronal viability 24 h post electroporation with AX004 antibody. Non-pycnotic nuclei were counted as viable, as determined by Hoechst 33358 staining (1 μg/mL) and expressed as a percentage of total (*n* = 2 independent experiments in hexaplicate). Data are presented as mean ± SEM. No statistically significant change between groups stated as ns. **d** Primary neurons 24 h post electroporation (MaxCyte Technology) with AX004 (60 μg) were lysed and intracellular ATP levels were measured with CellTiter-Glo® Luminescent Cell Assay (Promega) based on manufacter’s instructions. The experiment was repeated from 2 independent preparations in triplicates. Data are represented as arbitrary units (AU) and shown as mean ± SEM. No significant change between groups stated as ns

We next investigated whether the therapeutic candidate DC8E8 has any harmful effects on neurons in vitro. Since the antibody is not uptaken into neurons spontaneously, cells were transfected with fluorescently labelled DC8E8 antibody (Alexa Fluor 546) using MaxCyte electroporation. Already at 2 h after electroporation DC8E8 antibody decorated microtubules inside neuroblastoma cells SH-SY5Y (Fig. 3 a), thus confirming that the antibody molecules are functional and retained their ability to bind tau. We then examined the effect of DC8E8 antibody on neuronal viability at 24 h after electroporation using Hoechst staining (Fig. 3 b, c). Viability, here expressed as percentage of non-condensed nuclei in neurons with intracellular localisation of fluorescent antibody, was not affected when comparing neurons transfected with DC8E8 or control antibody (Fig. 3 b, c). We also assessed physiological functions of neurons at 24 h after electroporation of DC8E8 into cells. We did not detect any significant change in ATP levels between neuronal populations electroporated with control or DC8E8 antibody (Fig. 3 d). Neuronal viability was also not affected when DC8E8 antibody was present extracellularly by its addition to the surrounding culture media (data not shown). In summary, these data confirmed that DC8E8 antibody did not interfere with viability or physiological functions of neurons, thus strongly supporting its safety properties also at a cellular level.

### Extracellular pathogenic tau species of various size and conformation are internalized by neurons in concentration and time-dependent manner

**Fig. 4:**
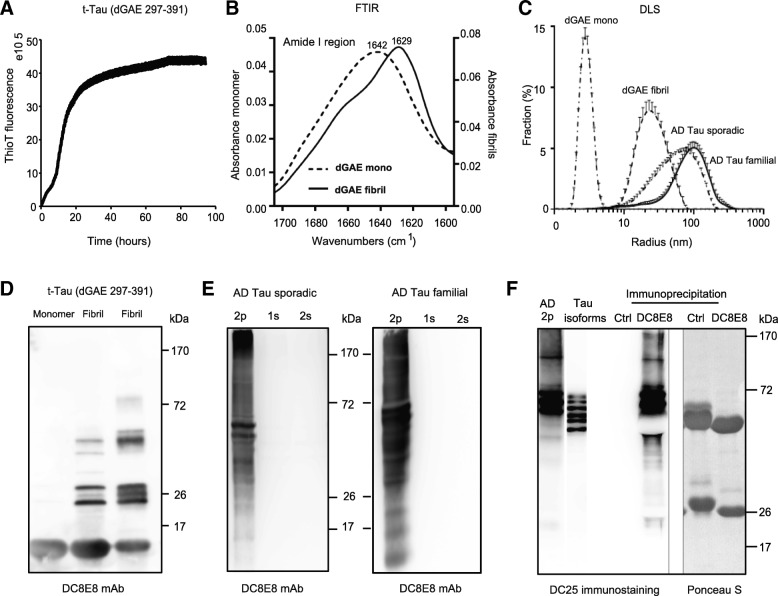
Characterization of different tau aggregates: truncated tau (dGAE), human AD brain-derived tau and ability of DC8E8 antibody to recognise various forms of diseased tau protein. **a** Kinetics of t-tau fibril formation was monitored over time by Thioflavin T fluorescence until it had reached plateau. t-tau indicates recombinant truncated PHF-core tau (dGAE, 297–391/4R). **b** FTIR spectroscopy confirmed a change in the structure after in vitro fibrillization of recombinant PHF-core subunit dGAE. The prevalent secondary structure changed from disordered to beta-rich structure. **c** Dynamic light scattering measurements of t-tau monomer (dGAE 297–391), heparin fibrillized t-tau, human brain-derived sporadic and familial AD tau (sarkosyl-insoluble 2p fraction, sonicated). All three sonicated fibrillary tau species were composed of high-molecular particules with an average radius spanning from 20 to 100 nm. **d** Non-reducing SDS-PAGE using pan tau antibody DC25 confirmed formation of tau fibrils (297–391/4R). Tau (dGAE 297–391) monomer has been used as a control representing band of one specific size (1 μg, band runs at 10–15 kDa). Heparin-assembled t-tau showed a pattern with more fibrillary species. **e** Western blot analysis of AD-tau preparations (sporadic and familial form) using microtubule binding domain DC8E8 antibody showed as individual fractions of sarkosyl-insoluble AD-tau preparations (2p, 1 s, 2 s). Both preparations of sarkosyl-insoluble fraction (2p) confirmed the presence of a wide range of assembled tau inclusions with DC8E8 antibody. **f** Sarkosyl-insoluble high-molecular weight tau isolated by centrifugation partially dissociates on the SDS PAGE and forms a typical A68 triplet. DC8E8 captured all diseased tau forms present in the sarkosyl extract from human AD brain (sporadic, Braak VI). The Western blot was developed with DC25 pan tau antibody. The panel on the right shows the same membrane stained with Ponceau S protein dye with the major bands corresponding to the chains of antibodies used in immunoprecipitation*.* Six recombinant tau protein isoforms were included for comparison

Tau protein in Alzheimer’s disease can form multiple different variants and species via the pathogenic process of misfolding [19]. The presence of pathogenic tau proteins actively released from diseased neurons in vitro and detected also in interstitial fluid (ISF) and CSF, clearly demonstrated the importance of extracellular tau species in the propagation of tau pathology [11, 62, 87, 96–100]. The spread of extracellular tau protein species with its initial step of tau neuronal entry [26, 95] has been shown to be regulated by tau quaternary structure [45, 74]. Formation of tau fibrillar filamentous species with cross-β-structure/core and morphology similar to NFTs can be potentiated in vitro [4, 6, 60]. The truncated tau protein (dGAE, 297–391), which encompasses the PHF-core region, assembles into PHF-like fibrils through the repeat domain as revealed by cryo-EM study [4, 23, 25]. Therefore, to investigate whether primary neurons can spontaneously internalize relevant pathogenic tau variants, we used the in vitro fibrillized form of recombinant truncated 95-amino acid tau fragment (297–391/4R) [4, 82, 94]. To cover the whole spectrum of truncated oligomerized and otherwise modified tau species (tauons) present in AD brain, we also prepared sarkosyl-insoluble fractions (2p) from sporadic and familial human AD brains (Braak stage VI). AD tau aggregates contain a compact core rich in the cross-beta structure [25]; using Thioflavin T (ThT) fluorescence, infrared spectroscopy and dynamic light scattering, we characterized the assembly of in vitro pre-formed recombinant truncated tau fibrils in terms of internal structure and size (Fig. 4a-d). Development of ThT fluorescence during assembly reaction indicates increasing content of beta structure, which eventually reached a plateau (Fig. 4a). FTIR spectroscopy of truncated tau fibrils (297–391/4R), compared with those of monomeric truncated tau, revealed nearly complete disappearance of a broad absorption maximum characteristic of disordered monomer (1642 cm^− 1^) and creation of a sharp maximum at 1629 cm^− 1^, which can be ascribed to the beta-sheet component of amide I region [79] (Fig. 4b). Characterization of all three different preparations of tau (fibrillized truncated tau, AD sporadic and AD familial tau) by DLS confirmed the presence of a wide spectrum of tau pathogenic species varying in size (with 20–100 nm radii) (Fig. 4c) and conformations (Fig. 4b, c, d).

Finally, we analysed if therapeutic candidate antibody DC8E8 can recognise the set of prepared tau species (recombinant truncated tau, human derived AD sporadic tau and AD familial sarkosyl-insoluble tau). Indeed, we confirmed the ability of DC8E8 antibody to capture the entire range of pathogenic tau species of different sizes (tau aggregation intermediates, fibrils and both low- and high-molecular-weight oligomers, multimers comprising all stages of tau protein oligomerization) and origin (Fig. 4d, e). Recognition of a complete panel of pathogenic tau protein oligomers and aggregates is an important prerequisite for their effective neutralization by a candidate therapeutic antibody. Therefore, we have also examined DC8E8 binding to sarkosyl-insoluble high-molecular-weight AD tau extract that had been immunoprecipitated after a gentle sonication, maintaining a native status of tau oligomers. We have compared western blot profile of the 2p sarkosyl extract of the AD brain with the DC8E8 immuno-precipitated material of the same, using a pan tau, repeat-region specific monoclonal antibody DC25 (tau 347–353) [106]. DC8E8 captured all tau species present in the extract, whereas an antibody with an unrelated specificity DC51 did not capture any tau-reactive material (Fig. 4f). Therefore, in addition to binding monomeric pathological tau proteins [49], DC8E8 also recognizes a full spectrum of high-molecular weight pathological tau.

**Fig. 5:**
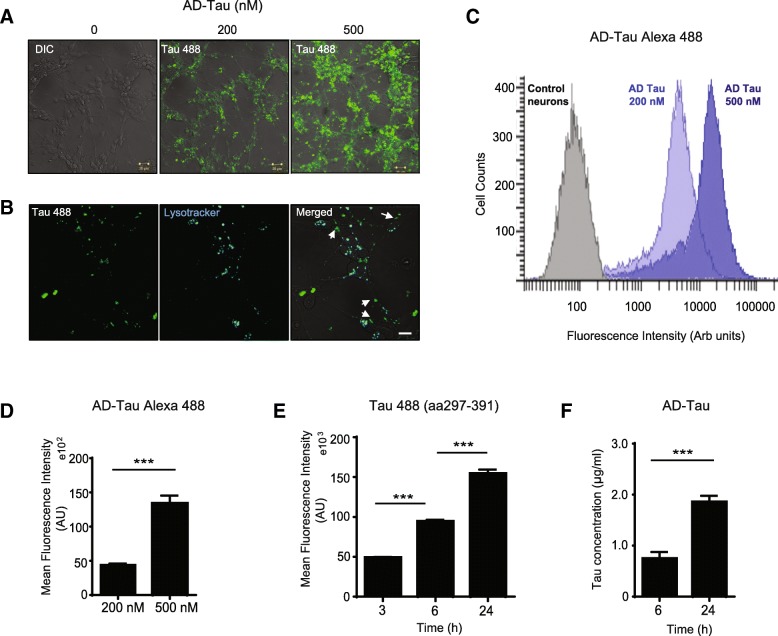
Pathogenic tau species efficiently enter primary neurons in concentration- and time-dependent manner. **a** Representative image of neuronal uptake of extracellular human-derived AD tau (sarkosyl-insoluble 2p) fluorescently labelled with Alexa Fluor 488. Neurons (2–4 days in vitro) were incubated with 200 nM or 500 nM AD-tau Alexa Fluor 488 for 24 h. Images were obtained using LSM 710 confocal microscope (DIC grayscale, AD tau Alexa 488 green). Scale bar, 20 μm. Panel **b** shows intracellular localization of tau Alexa 488 protein aggregates (100 nM) 24 h after addition to primary neurons cultured for 4 days in vitro*.* Tau Alexa 488 oligomers (green) are partially engulfed in lysosomes stained with a fluorescent dye LysoTracker (75 nM) indicated by co-localization in merged image (appears in cyan signal). Cytoplasmic “free” AD tau (green) is indicated with white arrows. Scale bar 20 μm. **c)** Quantification of relative fluorescence intensity (arbitrary units) utilizing flow cytometry for internalized AD tau Alexa 488 (200 nM and 500 nM, 24 h) in primary neuronal cells. A right shift (blue - dark blue) in the distribution indicates a concentration-dependent increase in the amount of internalized AD tau in neurons (increase in fluorescence). Control neurons (no AD tau) indicated in grey. **d** Flow cytometry analysis of AD tau Alexa 488 neuronal uptake with increasing concentrations (200 nM; 500 nM after 24 h). Data are presented as mean ± SEM. *p < 0.001, difference between 200 nM vs 500 nM AD tau Alexa 488 treated neurons. Experiments were performed in triplicates from 2 separate neuronal preparations. **e** Time dependent effect of t-tau Alexa Fluor 488 (297–391; 100 nM) neuronal internalization quantified at indicated time point (3, 6 and 24 h) by flow cytometry showed as mean fluorescence intensity (arbitrary units). Results were obtained from 2 separate cultures and data are presented as mean ± SEM. *p < 0.001, difference between 3 h and 6 h; and between 6 h and 24 h after t-tau Alexa 488 treated neurons. In flow cytometry experiments a minimum of 10.000 events per sample was analyzed. **f** Neuronal uptake of AD tau 2p fraction in cells harvested at indicated time points (6 h and 24 h) was evaluated by a sandwich ELISA (DC25/DC190). Data were corrected for protein concentration in the sample. Increase in the amount of intracellular AD tau in cell lysate pellets in time was detected. Experiments were performed in 2 independent preparations. *p < 0.001, difference between 6 and 24 h

To ensure that the prepared species can act as tau seeds and their size does not exceed what neurons are able to uptake, the aggregates were sonicated before administration to cell media. We next incubated fluorescently labelled human brain-derived sarkosyl insoluble tau (2p) with murine primary neurons at two increasing concentrations. As examined by live cell confocal imaging (Fig. 5a, b), extracellular AD tau species were readily internalized in primary neurons and were trafficked to lysosomes which futher confirmed their intracellular localisation (Fig. 5b). However, not all the up-taken tau proteins were engulfed in the lysosomes, part of them were present in the “free” cytoplasmic fraction. We suggest that this fraction of tau could serve as a seed for the aggregation of endogenous tau and further propagation of tau pathology. We found that sarkosyl-insoluble AD tau species were internalized in primary neurons in a concentration-dependent manner which is in accordance with previous reports using tau P301S variant [21]. Moreover, neuronal internalization of fibrillized truncated tau and AD tau evaluated by different independent methods (flow cytometry, tau specific ELISA, immunocytochemical colocalisation of internalized AD tau with human tau antibody HT7 and Western Blot), resulted in a statistically significantly increased neuronal uptake of these pathogenic tau species over time (Fig. 5e, f, Additional file 1: Figure S1a, b). Thus, disease relevant tau species in our experiments, covering the whole range of tau variants of different size and origin (Fig. 4b-d) are readily internalized into primary neurons in vitro (Fig. 5 a – e; Additional file 1: Figure S1 a, b).

### Therapeutic antibody DC8E8 prevents neuronal internalization of disease-relevant AD tau variants

**Fig. 6:**
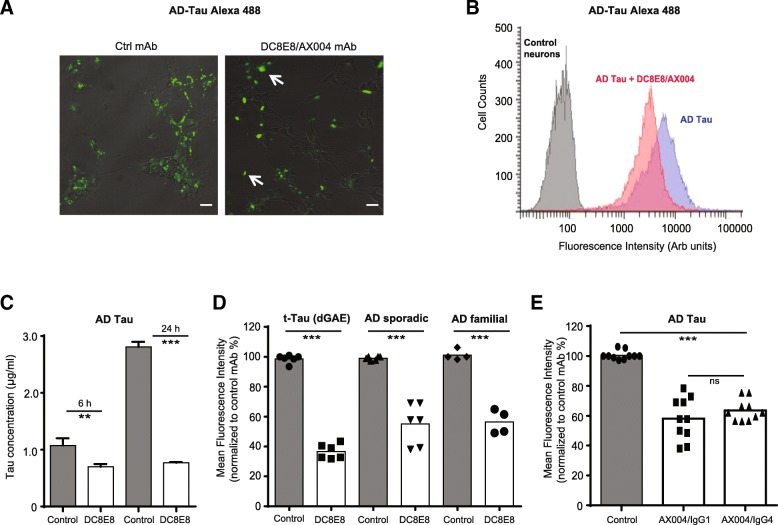
Therapeutic candidate antibody DC8E8 inhibits neuronal internalization of various extracellular diseased tau species. **a** Confocal microscope images of AD tau fluorescently labelled with Alexa 488 (100 nM, 24 h) in the presence of irrelevant control mAb DC51 or therapeutic DC8E8 antibody (both green). AD tau Alexa 488 in pre-formed complex with DC8E8 (15 min incubation, 37 °C) attenuated intracellular localization of AD tau in neurons in comparison to cells incubated with control mAb (AD tau Alexa 488 present intracellularly). Scale bar, 20 μm. **b** Flow cytometric quantification of mean fluorescent intensity (arbitrary units) of AD tau Alexa 488 (100 nM, 24 h post addition to neurons) in the presence of control and DC8E8 or AX004 antibody (168 μg/mL ~ 1 μM). A left shift (blue – red) indicates DC8E8-mediated inhibition of AD tau internalization into neurons. Control neurons with no AD tau are indicated in grey. **c** Neuronal internalization of AD tau at 6 and 24 h in combination with control or DC8E8 antibody (1 μM) analysed by a sandwich ELISA. Data were corrected for protein concentration in samples. Decrease in the amount of intracellular AD tau in neurons was measured with DC8E8 antibody. Experiments were performed in 2 independent cultures. *p < 0.001, difference in 6 and 24 h detected between AD tau with Control versus AD tau with DC8E8 antibody. **d** Quantification of neuronal uptake of various pathological tau species: recombinant truncated tau (dGAE, 297–391/4R) and human brain-derived sporadic and familial AD tau (100 nM for 24 h) in the presence of Control or DC8E8 antibody (both 1 μM). Experiments performed from 2 independent measurements are represented as mean fluorescence intensity normalized to % of the control antibody. For flow cytometry experiments a minimum of 10.000 events per sample was analysed. DC8E8 antibody strongly blocked the neuronal uptake of all three tau species (truncated tau, AD sporadic and AD familial tau) compared to tau species with Control antibody. Data are presented as mean ± SEM. Statistically significant change between groups stated as ***p < 0.001. **e** Comparison of AX004 antibody of isotypes IgG1 versus IgG4 (1 μM) in slowing neuronal internalization of AD tau Alexa 488 (100 nM, sonicated 2 min) after 24 h. Summary of data measured by flow cytometry from 5 independent experiments (*n* = 10). All data are presented as mean +/− SEM. Statistically significant change between AD tau with Control antibody in comparison to AD tau with AX004 of both isotypes (IgG1; IgG4). Statistically non-significant change shown as ns

Recently, in vitro functional assays of tau neuronal uptake and seeding have been used to screen for the best candidate antibody to ensure maximal therapeutic efficacy [14, 15, 68, 88]. In these studies, mostly antibodies targeting the mid domain of tau showed the highest capacity to block tau seeding in various in vitro and in vivo models in direct comparison to N- or C-terminal targeting antibodies [15, 68, 88]. Monoclonal antibody DC8E8 binds to MTBR at four highly homologous sites [49] and can recognise various forms of pathogenic tau (Fig. 4 d, e). DC8E8 therapeutic antibody is not internalized into tau diseased neurons (Fig. 2) and predominantly mediates its mechanism of action extracellularly. Therefore, we next investigated whether DC8E8 and its humanized version (AX004/IgG1), can also capture and neutralize various extracellular pathological tau variants and their neuronal internalization (the initial step of seeding). To do so, we tested the potency of DC8E8 antibody to reduce the uptake of extracellular diseased AD tau into primary neurons. We examined the amount of fluorescently labelled AD tau internalized to neurons in the presence of control versus DC8E8 antibodies using confocal microscopy in combination with flow cytometry and ELISA. Indeed, AD tau fluorescent punctae accumulation within neurons was statistically significantly reduced in the presence of DC8E8 antibody, with larger fluorescent aggregates present outside the cells in media (Fig. 6 a). Presumably, AD tau in the presence of DC8E8 antibody formed complexes that were primarily localized outside the neurons (Fig. 6 a). Under these conditions, DC8E8 antibody potently blocked the uptake of sporadic sarkosyl-insoluble AD tau already at 6 h after addition to the culture media with a more pronounced effect after 24 h of incubation (Fig. 6 c).

Previous reports and our proteomic data (not shown) suggest the existence of heterogeneous pool of tau variants present in Alzheimers disease brain comprised predominantly of various truncated AD-tau species [35, 44, 51, 63, 104]. Therefore, to gain a more complex insight we analysed whether DC8E8 antibody can prevent internalization of different relevant pathogenic tau variants present in AD human brain (PHF-core truncated tau, AD sporadic and AD familiar tau). DC8E8 antibody proved to be a strong potent blocker of tau neuronal uptake for all tau species examined (Fig. 6 d). This is a direct evidence that DC8E8 antibody recognises a whole set of pathogenic tau variants (Fig. 4) and is concominantly capable of capturing the majority of extracellular tau by forming complexes with them, and thus preventing their neuronal internalization.

Ultimately, we investigated whether the antibody isotype is actively involved in mediating the blocking effect of AD tau uptake into neurons. For this purpose, we used humanized version of DC8E8 antibody (AX004) of two different isotypes, AX004/IgG1 or AX004/IgG4, as these two isotypes have been used for all tau-targeted immunotherapies in clinical development since 2013 [71]. We found that both isotypes of AX004 antibody exhibited a pronounced inhibitory effect on the internalization of AD tau into neurons (approx. by 50%, Fig. 6 e). Notably, both AX004 isotypes, IgG1 and IgG4 attenuated AD tau neuronal uptake to a similar extent with no statistically significant difference between them (Fig. 6 e).

### Antibody DC8E8 potently abrogates entry of human derived AD tau into neurons via masking tau recognition sites to heparan sulfate proteoglycans (HSPGs)

**Fig. 7:**
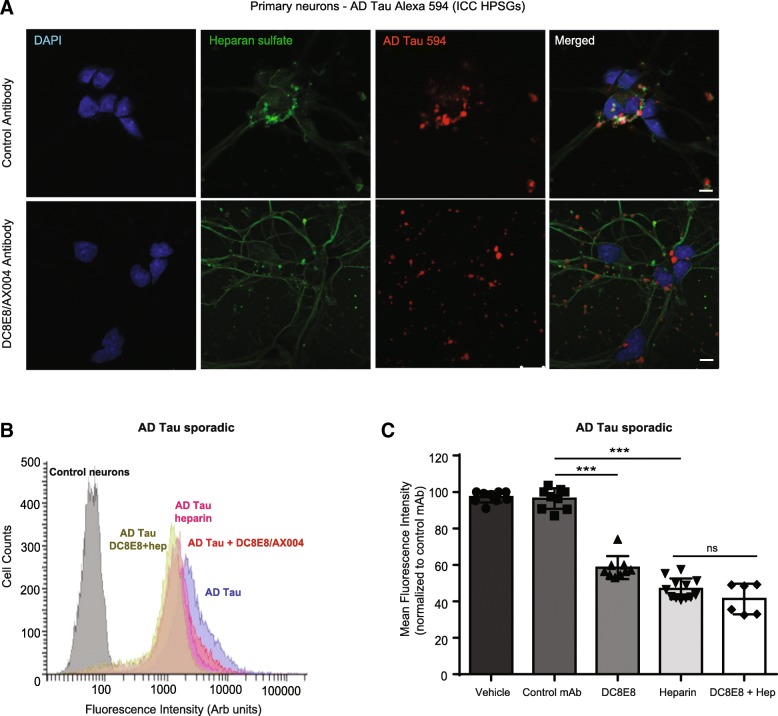
DC8E8 targeting MTBD inhibits AD tau neuronal uptake via masking binding sites to heparan sulfate proteoglycans. **a** Co-localization of sarkosyl-insoluble AD tau PHFs and heparan sulfate in primary neurons. AD tau PHFs were fluorescently labelled with Alexa Fluor 594 (red) and added to cultured media. After 20 h neurons were fixed and immunostained with antibody against heparan sulfate (green, 10E4). Neurons were counterstained with DAPI (blue, nuclei) and confocal microscopy images were captured. Scale bar, 10 μm. **b** Representative distribution of mean fluorescent intensity (arbitrary units) in neurons treated with AD tau Alexa 488 (100 nM) for 24 h in the presence of control antibody DC51 (blue), DC8E8 antibody (red), heparin alone (pink) or heparin in combination with DC8E8 (beige) measured by flow cytometry. A left shift (blue – pink) indicates decrease in AD tau neuronal internalization in the presence of heparin (implies requirements of heparan sulfate proteoglycans) to similar extent as DC8E8-mediated inhibition of the internalized AD tau (red). Non-significant difference in the distributions of heparin alone (pink) and heparin with DC8E8 (beige) was detected. Non-treated Control neurons are indicated in grey. **c** Quantification of AD tau Alexa 488 internalization (100 nM, 24 h) in the presence of Control or DC8E8 antibody (1 μM), heparin (5 μM) or heparin with DC8E8 determined by flow cytometry. Experiments were performed in duplicates with a minimum of 3 independent measurements and expressed as mean ± SEM. Statistically significant change between groups stated as *p < 0.001*.* Flow cytometry experiments include a minimum of 10.000 events per each sample showed

An important hallmark of AD and related tauopathies is the formation of various pathogenic tau variants with strong ability to spread to neuro-anatomically connected regions in the brain [17]. This process involves the release of proteinaceous seeds into the extracellular space and their uptake by neighbouring cells where they induce fibrillization of intracellular tau [26, 95]. Recent data firmly established the critical role of sulfated heparan proteoglycans in the internalization and release of tau from neurons depending on the aggregated state of tau and its quarternary structure [21, 37, 46, 74, 101]. Pathogenic tau protein variants of specific size and conformation contain several heparin binding motifs predominantly in the microtubule-binding repeats domain (MTBR) important for neuronal internalization [27, 37, 80, 103]. DC8E8 is an anti-tau antibody interacting with 4 different epitopes with homologous amino acid sequence, also localised in each of the repeats of MTBR domain [49]. Therefore, we challenged the hypothesis that the formation of a complex between therapeutic antibody and pathogenic AD tau masks the HSPG binding sites, thus preventing its neuronal entry. Initially, we examined the involvement of HSPGs in sarkosyl-insoluble AD tau in neuronal internalization process. As shown above (Fig. 5 b), the intra-cellular localization of tau has been supported by its colocalization with lysosomes. Here, we detected colocalisation of numerous punctae of AD tau amyloid fibrills with heparan sulfate present on the surface of neurons (Fig. 7 a). Conversely, when therapeutic DC8E8 antibody was present in the culture media, AD-tau fibrils were not internalized in neurons and no co-localization with heparan sulfate was detected (Fig. 7b). It has been shown by others, that heparin can efficiently block the interaction of tau with HSPGs [37, 103]. Indeed, in the presence of heparin (5 μM) AD tau failed to bind to neurons and its internalization was potently inhibited (Fig. 7c). The effect of therapeutic antibody DC8E8 or heparin alone on blocking of neuronal AD tau uptake were comparable. We speculate that the slightly higher efficiency of blocking with heparin alone over DC8E8 can be mediated by the presence of other amyloid protein aggregates present in crude sarkosyl-insoluble AD tau fraction. Most importantly, no statistically significant additive effect was observed when neurons where treated with heparin alone or with heparin in combination with DC8E8 antibody (Fig. 7c).

**Fig. 8:**
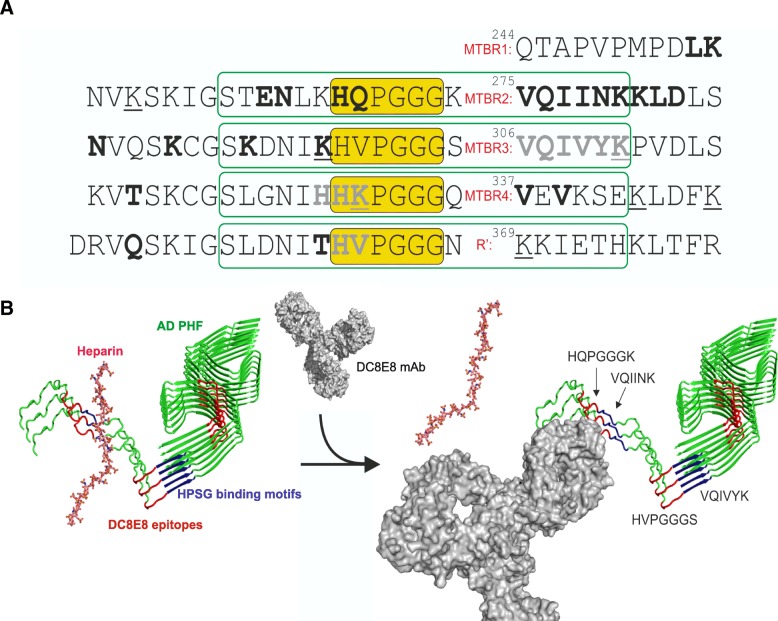
Antibody blocks neuronal internalization of human AD tau by masking HSPGs recognition sites via steric hindrance. **a** Proposed mechanism of action of therapeutic candidate antibody DC8E8 and its humanized version AX004. Antibody binds to tetratop in the microtubule binding region (MTBR) with a common amino acid pattern HxPGGG (yellow boxes). AD tau contains several heparan sulfate proteoglycan binding sequences that are present in close proximity to DC8E8 tetratop recognition site. Here shown based on determined interaction of heparin with either four repeat MTBR (bold) or with three repeat MTBR (grey bold) [103]. Additionally, lysine residues responsible for interaction of full-length tau with heparin are presented as underlined. Particular strong interaction was identified for lysine-rich region downstream of R4 [27, 80]. We hypothesize that the binding of antibody affects the tau residues in the vicinity of its epitopes (green boxes). **b** Soluble heparin chain (stick model, pink carbon atoms, PDB ID: 3IRJ) is bound to the fuzzy coat of the PHF core (green, modified according PDB ID 5O3L) [25] along its binding motifs on tau represented by hexapeptides ^275^VQIINK^280^ and ^306^VQIVYK^311^ (highlighted in blue, as arrows) [27, 80]. The interaction of DC8E8 antibody (gray surface model) with disordered part of PHF tau (epitopes R1 and R2 highlighted in red) blocks adjacent heparin binding motifs. Such arrangement inhibits neuronal internalization by DC8E8-mediated blocking of the interaction between heparan sulfate binding motifs on tau to neuronal surface HSPGs via steric hindrance. For three tau chains, fuzzy-coat disordered repeats R1 and R2 are shown. All molecules are presented in full proportion. Model of Pembrolizumab, a full length IgG4 antibody (PDB ID 5DK3) was used as a proxy for DC8E8 structure

Based on our previous structural data of DC8E8 antibody binding epitopes localised in MTBR (R1-R4) and, embracing the conserved sequence motif HxPGGG present four times on tau protein, we propose the mechanism of how DC8E8 can block AD tau neuronal internalization (Fig. 8 a, b). Multiple HSPGs binding sequences on tau, predominantly localized in R2 and R3 (residues 274–335) and close to lysine-rich region after R4 repeat domain [27, 49, 80, 103] are all in close proximity to DC8E8 antibody binding epitopes: ^268^HQPGGG^273^ (R1), ^299^HVPGGG ^304^ (R2), ^330^HKPGGG ^335^ and ^362^HVPGG ^367^ (detailed residue level description of HSPG binding sites from NMR data described on Fig. 8 a). Therefore, formation of a complex between DC8E8 antibody and extracellular AD tau reduces the number of available heparan sulfate binding motifs, which interferes with the mode of HSPG-tau interaction and at the same time inhibits further aggregation. Overall, tau sequence analysis data in combination with the presented biological functional analysis, strongly suggest that the unique characteristics of the epitope of DC8E8 antibody determine its therapeutic mode of action as the ability to effectively block tau beta-structure formation and AD tau neuronal internalization by masking HSPG recognition sites via steric hindrance (Fig. 8 a, b).

## Discussion

Immunotherapy targeting the major proteinaceous pathological lesions currently represents the main approach in the search for an AD modifying treatment. Given its critical role in the pathogenesis of AD, and considering the strong correlation of NFTs pathology with cognitive decline of patients, diseased tau proteins have become a very promising molecular target for disease-modifying immunotherapy of AD. Therefore, we have selected a monoclonal anti-tau antibody DC8E8 with a unique tau binding mode consisting in recognition of four homologous epitopes present in each of the repeats of MTBD [44, 49, 72, 73]. Importantly, tau structural determinants characterized by DC8E8 antibody were utilized also for the development of an active vaccine AADvac1 (currently in the clinical trials), which contains one of the DC8E8 epitopes [73]. Similar to active vaccine AADvac1, passive immunotherapy with DC8E8 antibody confirmed its strong efficacy in prevention of the development of tau pathology [49, 50]. Serum antibodies generated after vaccination with AADvac1 and DC8E8 antibody target the same epitopes on tau, exhibit a strong preference for pathological tau over full-length tau [70] and display similar efficacious effects in animals expressing pathogenic truncated tau, indicating that both active and passive immunizations share a common mechanism of action.

Detection of intracellular tau protein extracellularly in interstitial space or CSF in AD patients [41, 97–99] and controversy in studies examining intracellular [13, 33, 81] vs extracellular mode of action of antibodies, shifted most of the current research to screen for antibodies targeting predominantly extracellular tau species [36]. Using ex vivo neuronal culture models we were able to show that DC8E8 antibody was not readily internalized in neurons with diseased tau expression even several hours after addition to culture media, suggesting extracellular mechanism of action as the main factor contributing to its efficacy. This is in a line with other preclinical studies where despite profound efficacy antibodies where not internalized into neurons [102]. However, we could not completely exclude the neuronal uptake of a small amount of DC8E8 antibody in complex with tau aggregates as found by others [21], which could be either under our detection threshold or due to ex vivo cell culture conditions used which might differ from in vivo conditions. Additionally, Alzheimer’s tau protein forms toxic complexes with cellular membranes of primary hippocampal cultures [3], which can then potentially mediate neuronal uptake of the antibody. Therefore, we examined the binding properties and toxicity of DC8E8 antibody also if present intraneuronally. In ex vivo conditions we electroporated fluorescently labeled DC8E8 antibody into the cytosol of primary neurons. Binding of DC8E8 to microtubule-associated tau proteins was detected very early after electroporation showing that delivered antibody molecules were active. The internalized DC8E8 antibody did not interfere with the viability or physiology of neurons even at later time points. Thus, although DC8E8 antibody preferentially binds to pathogenic tau [49], it can interact with physiological tau but obviously does not interfere with its normal function. These results also support our recent findings from the phase I clinical trial of active AADvac-1 [70, 73]. The vaccine, which contains one of the DC8E8 epitopes and thus elicits production of DC8E8-like antibodies, showed an exceptionally favourable safety profile. Hence, both passive and active DC8E8-based immunotherapeutic approaches thus appear safe.

In recent years, tau pathophysiology has been necessarily linked with findings that tau can propagate across neuro-anatomically concected brain regions histologically defined as Braak staging [8, 12]. Trans-neuronal propagation of tau aggregates involves the release of proteopathic seeds into the extracellular space with more free tau entering recipient neurons and initiating further fibrillization. The predominantly extracellular tau protein is thus a readily accesible target for therapeutic antibodies. The efficacy of antibodies depends on the epitope selection and ability to recognise relevant pathogenic tau species responsible for trans-neuronal transmission. In this work we demonstrated the ability of DC8E8 antibody to recognise a heterogeneous pool of pathogenic tau aggregates of various sizes (LMW, HMW), conformations (native status of tau multimers), and origins (recombinant PHF-core truncated tau, sporadic and familial AD), making this antibody a suitable therapeutic candidate. These data were confirmed by an independent study showing that an antibody that binds HxPGG motifs, present four times in MTBD, binds all human PHF tau variants [88]. Furthermore, clinical potential of DC8E8 was highlighted by our previous finding where we demonstrated that DC8E8 has a strong binding preference for truncated diseased tau protein (151–391/4R) over the physiological full-length tau monomer 2N4R [49], thus overcoming the concerns of harmful effects linked to potential elimination of healthy physiological tau [59].

Another challenge discussed in various studies are the unclear/unknown properties of pathogenic tau species responsible for neuronal toxicity and disease progression in AD brains [19, 42, 64, 86, 87]. Therefore, to supply a wide range of pathogenic tau species naturally present throughout course of Alzheimer disease, we prepared oligomerized and aggregated tau species (sarkosyl-insoluble fraction 2p) from sporadic as well as familial forms of AD (stage Braak VI) [85]. Each fraction was further sonicated to maximize the variability in size and conformations of individual tau species present in AD brains. Indeed, the strong spreading potency in vivo in animals has been demonstrated also using human-derived seed-competent sarkosyl-insoluble tau fractions as inoculum [35, 42, 83, 88].

Parallel with our previous findings, disease associated tau in post-mortem AD brains, appears to contain large amount of truncated tau fragments [18, 51, 69, 72, 88, 92, 93, 104, 106]. Tau fragments truncated at both N- and C- termini that contain MTBD are more prone to aggregation [51], with tau fragment terminated at Glu 391 in vitro into a cross-β core through the repeat domain into PHF [1, 91]. Thus, as an alternative to the natural truncated tau variants present in AD brains, here we also tested a recombinant truncated tau fragment dGAE encompassing amino acids 297–391 of full length tau that corresponds to the PHF-core region [4, 25]. AD-relevant tau variants tested here were readily spontaneously internalized by primary neurons in time and concentration-dependent manner. Similar results have been reported recently, however using other experimental conditions: different tau variants (full-length 2N4R tau fibrils [27], full-length tau with P301S mutation, the cause of familial form of frontotemporal dementia (FTD) rather than AD [21]), or utilizing a non-neuronal cell based seeding assays [88] which requires facilitation of tau seeding with additional protein carriers (lipofectamine) [15].

The central role of misfolded tau aggregates, actively released from diseased neurons to extracellular space, has been demonstrated in the process of trans-neuronal propagation [36, 77]. Therefore, targeting extracellular tau by antibody with the ability to recognise distinct pathogenic tau variants may efficiently abrogate spreading of tau pathology and thus represent an efficient therapeutic strategy. Different sets of cellular in vitro functional assays examining tau neuronal seeding and propagation have been used to identify new immunotherapeutics with a potential maximum efficacy [15, 68, 88, 102]. Here we tested the ability of a candidate therapeutic antibody DC8E8 to block neuronal internalization of pathogenic tau species, thereby slowing down inter-neuronal propagation of tau pathology. Indeed, antibody DC8E8 and its humanized version AX004 were both highly effective in neutralization of the initial uptake of various disease-associated tau seeds. Recently, testing different sets of antibodies showed that only antibodies with epitopes localized in mid-region of tau protein, and not at the N-terminus, displayed the highest potential in tau seeds depletion/neutralization [15, 68, 88]. We also confirm here that DC8E8 antibody with its epitope localized in the aggregation-prone MTBR domain has a strong tau uptake neutralization ability, even though the decrease in fluorescence intensity was not complete. We cannot exclude the contribution of other tau-unrelated protein aggregates present in crude sarkosyl-insoluble AD brain material and potentially different sensitivity of our approach when compared to aggregation-biosensor HEK293T cell lines of non-neuronal origin [38]. Importantly, another antibody PT83, with highly similar binding pattern as DC8E8, localised in all four repeats of MTBD with xxxPGG motif, showed almost complete inhibition of tau seeding into non-neuronal HEK293 FRET cells over-expressing truncated tau fragment 243–375 [88]. In the same study, another MTBD antibody tested in vivo for neutralization of tau seeding exerted a strong inhibition in transgenic mice. Conversely, first-generation therapeutic N-terminal antibodies selected based on their affinity were significantly less active in functional seeding assays and unable to recognise terminaly truncated tau fragments in isolated PHFs [88]. The fact that PHFs from AD brains contain truncated tau variants at either one or both N- and C- termini [18, 72, 106], together with an assumption that AD tau fragments contain at least part of an intact MTBR, predisposes DC8E8 antibody with its unique binding tetratop located throughout this region as a promising therapeutic candidate targeting pathogenic (seed-competent) tau species.

Tau aggregates bind heparan sulfate proteoglycans (HSPGs) on the neuronal surface, promoting tau neuronal uptake and seeding via macropinocytosis, thereby transmitting tau pathology among neurons [37, 74]. Increased amount of sulfated proteoglycans in AD brain [54], colocalisation with neurofibrillary tangles [29], and recently identified involvement of HSPGs in the process of tau neuronal internalization [27, 37, 57, 74, 84, 101] and tau secretion [46] are indicative of critical role of HSPGs in the transmission of tau pathology among neurons. Neuronal internalization of aggregates by HSPGs has been shown to be dependent on aggregate size, conformation [27, 41, 74, 84] and the presence of specific heparin binding motifs on tau responsible for tau/HSPG interaction [20, 27, 80, 103]. Here we demonstrated that DC8E8 antibody can not only recognise various pathogenic tau variants, but has the ability to abrogate tau neuronal entry. Knowing that binding motifs responsible for tau/HSPGs interaction are mainly localized in the same microtubule binding repeat domain as the epitopes of therapeutic candidate antibody DC8E8, we analysed in detail if the formation of a complex between tau aggregates and antibody can potentially mask recognition site on tau important for neuronal internalization process. By employing AD-derived tau aggregates which are more likely to be internalized via HSPGs [27], as confirmed here by co-localization of heparan sulfate and internalized AD tau aggregates, we also tested the ability of DC8E8 antibody to inhibit tau neuronal internalization alone or in combination with heparin. These in vitro functional experiments strongly support the hypothesis that the binding of DC8E8 antibody to AD tau most probably masks HSPGs binding motifs on tau, as neuronal entry of tau was abrogated to a similar extent. Interventions targeting the binding of tau aggregates to neuronal sulfate proteoglycans have been suggested as a new therapeutic opportunity [27, 37, 46, 74]. Therefore, to achieved detailed mechanistic insight, we further performed analysis of binding properties of DC8E8 antibody to pathogenic tau protein. Overall, positively charged lysines, arginine, and histidine are the important spots in binding between tau and heparin as determined by NMR titration [66, 80, 103]. Moreover, hexapeptides presents in the second (R2) and third repeat domains (R3) of MTBD on tau: (^275^VQIINK^280^ and ^306^VQIVYK^311^) defined as aggregation-prone and associated with PHF-core [25, 78, 89, 90], were also identified as heparin binding motifs [27, 66, 103]. Notably, the first two epitopes of therapeutic candidate antibody DC8E8 mapping residues 269–273 and 299–304 on tau are localised in close proximity to HSPG binding sequences on tau [49, 72]. Furthermore, a lysine rich region downstream of R4 has been also suggested as HSPG binding sequence [27, 80] again in proximity to DC8E8 binding to epitope 362–367 present in R4. Similar potential mechanisms of monoclonal anti-tau antibodies decreasing pathology in vivo were also suggested recently [27, 102]. Antibody HJ9.3 recognising epitope 306–320 in MTBR, similar to DC8E8 binding epitope, was found to be the only antibody tested with the unique ability to inhibit internalization of repeat-domain tau fibrils and AD tau aggregates in primary neurons with proposed potential mechanism of blocking tau/HSPG interaction [27, 48].

Taken together, analysis of tau binding properties of DC8E8 antibody together with the presented functional ex vivo cellular assays strongly indicate that DC8E8 antibody targeting four different epitopes in the MTBR domain of pathogenic tau, has unique therapeutic attributes with several mechanisms of action: 1) DC8E8 binding to tau protein precedes β-forming motifs which leads to inhibition of aggregation of pathogenic tau [49], 2) can recognise pathological tau variants of distinct sizes and origins (sporadic, familial AD) with preferential binding to pathogenic over physiological tau and most importantly 3) effectively inhibits spreading of tau pathology by interfering with AD tau neuronal uptake through masking several HSPG recognition sites via steric hindrance. Additionaly, DC8E8 antibody recognises repeat domain of tau even in its oligomerised form (confirmed by inhibition of neuronal uptake of dGAE tau oligomers), thus showing the exceptional ability of DC8E8 to bind to seeding-capable forms of tau. In conclusion, our results highlight a unique therapeutic mechanisms involved in active and passive approaches based on anti-tau DC8E8 antibody and its remarkable tau epitope used for the design of first-in man AADvac1 vaccine currently in Phase 2 human clinical trials [73], thus representing an exciting period in AD therapy.

## Additional file

Additional file 1: **Figure S1.** Human AD brain-derived tau protein internalized in primary neurons. **a)** Immunocytochemical co-localization of sarkosyl-insoluble AD tau PHFs and HT7 antibody in primary cortico-hippocampal neurons. AD tau PHFs were fluorescently labelled with Alexa Fluor 488 (green) and added to neuronal cultured media. After 24 h neurons were fixed and immunostained with antibody against human tau (red, MN1000 ThermoFisher). Neurons were counterstained with DAPI (blue, nuclei) and confocal microscopy images were captured. Scale bar, 10 μm. **b)** Western blot analysis of human AD tau (sarkosyl-insoluble 2p) internalized in primary cortico-hippocampal neurons over time. Experiments were repeated in triplicate with similar pattern obtained. (PDF 923 kb)
