# Supplementary figures and images for: Therapeutic antibody targeting microtubule-binding domain prevents neuronal internalization of extracellular tau via masking neuron surface proteoglycans
Source: Acta Neuropathol Commun. 2019 Aug 7;7:129. doi: 10.1186/s40478-019-0770-y (PMC6685285; doi:10.1186/s40478-019-0770-y)

## Supplementary Figure 1

### A AD Tau inside neurons

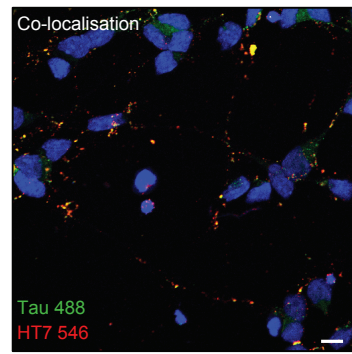

HT7 immunostaining

### B AD Tau neuronal uptake

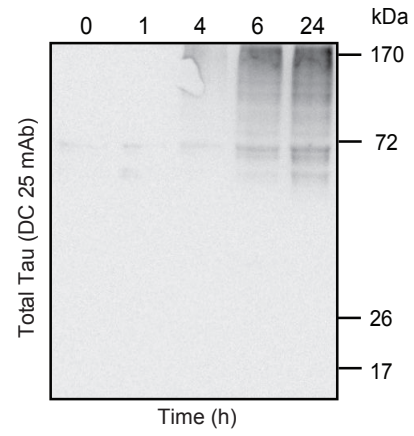

Supplement: Supplementary file 1 — Figure S1. Human AD brain-derived tau protein internalized in primary neurons. a) Immunocytochemical co-localization of sarkosyl-insoluble AD tau PHFs and HT7 antibody in primary cortico-hippocampal neurons. AD tau PHFs were fluorescently labelled with Alexa Fluor 488 (green) and added to neuronal cultured media. After 24 h neurons were fixed and immunostained with antibody against human tau (red, MN1000 ThermoFisher). Neurons were counterstained with DAPI (blue, nuclei) and confocal microscopy images were captured. Scale bar, 10 μm. b) Western blot analysis of human AD tau (sarkosyl-insoluble 2p) internalized in primary cortico-hippocampal neurons over time. Experiments were repeated in triplicate with similar pattern obtained. (PDF 923 kb) [file 40478_2019_770_MOESM1_ESM.pdf]
